# Impact of Sintering Temperature Variation on Porous Structure of Mo_2_TiAlC_2_ Ceramics

**DOI:** 10.3390/ma16165682

**Published:** 2023-08-18

**Authors:** Junsheng Yang, Yiquan Fan, Hua Tan, Wenkang Liu, Yijian Kuang, Xuejin Yang, Meili Cao, Jie Li

**Affiliations:** 1 College of Mechanical Engineering, Wuhan Polytechnic University, Wuhan 430048, China; yangjunsheng2008@163.com (J.Y.); fanyiquan1998@163.com (Y.F.); a2300120524@163.com (W.L.); kuangyijian1999@163.com (Y.K.); yangxuejin.2007@163.com (X.Y.); 13995577674@163.com (M.C.); 2 State Key Laboratory of Material Processing and Die & Mould Technology, Wuhan 430074, China; hua_tan@hust.edu.cn; 3 School of Materials Science and Engineering, Huazhong University of Science and Technology, Wuhan 430074, China

**Keywords:** Mo_2_TiAlC_2_, volume expansion rate, porosity, pore forming mechanism

## Abstract

Mo, TiH_2_, Al and graphite elemental powders were used as starting materials for the activation reaction sintering process, which was employed to fabricate porous Mo_2_TiAlC_2_. The alteration of phase constitution, volume expansion, porosity, pore size and surface morphology of porous Mo_2_TiAlC_2_ with sintering temperatures ranging from 700 °C to 1500 °C were characterized by X-ray diffraction (XRD), scanning electron microscopy (SEM) and pore size tester. Both the pore formation mechanism and activation reaction process at each temperature stage were investigated. The experimental results illustrate that the sintered discs of porous Mo_2_TiAlC_2_ exhibit obvious volume expansion and pore structure change during the sintering process. Before 1300 °C, the volume expansion rate and porosity increase with the increment of temperature. However, with the sintering temperature above 1300 °C, the volume expansion rate and porosity decrease. At the final sintering temperature of 1500 °C, porous Mo_2_TiAlC_2_ with a volume expansion rate of 35.74%, overall porosity of 47.1%, and uniform pore structure was synthesized. The pore-forming mechanism of porous Mo_2_TiAlC_2_ is discussed, and the evolution of pressed pores, the removal of molding agents, the decomposition of TiH_2_, and the Kirkendall effect caused by different diffusion rates of elements in the diffusion reaction are all accountable for the formation of pores.

## 1. Introduction

MAX phases are compounds with the general formula M_n+1_AX_n_ (n = 1–6), where M represents a transition metal, A represents a main group element, and X is either C or N [1,2,3,4,5]. In the MAX phase, the M atom and the X atom are combined by a covalent bond and ionic bond, and the M atom and the A atom are combined by a metal bond. Because of their distinct structure makeup, MAX phase compounds grant the characteristics of both ceramics and metals, including high modulus, low specific gravity, good thermal conductivity, machinability, thermal shock resistance, damage tolerance, thermal stability, creep resistance and oxidation resistance [6,7,8,9].

As further research on MAX phase materials is conducted, more and more new MAX phases have been investigated. In 2014, Liu et al. [10] first discovered and successfully prepared Cr_2_TiAlC_2_. Then, Caspi et al. [11] successfully prepared V_2_CrAlC_2_. The discovery of these new phases broadens the field of the MAX phase. Cr_2_TiAlC_2_ and V_2_CrAlC_2_ can be summarized as a kind of quaternary MAX phase (M′, M′′)_n+1_AX_n_, and the elements in the M position of this kind of quaternary MAX phase are composed of two transition elements, which may have better performance [12]. Since the new layered quaternary ceramic Mo_2_TiAlC_2_ was reported, its highly ordered structure has attracted a lot of attention [13]. Gao et al. [14] successfully synthesized Mo_2_TiAlC_2_. The elastic, thermodynamic and optical properties of Mo_2_TiAlC_2_ were studied by first-principles calculations, and the evidence of excellent stability of Mo_2_TiAlC_2_ was successfully obtained. Anasori et al. [15] prepared Mo_2_TiAlC_2_ by mixing and heating different elemental powder mixtures and found that it had stable structure and excellent electrical conductivity. Ma et al. [16] explored the structure, elastic and electronic properties, Debye temperature and theoretical Vickers hardness of Mo_2_TiAlC_2_ by first principles, and they pointed out that it had significant elastic anisotropy. At the same time, the corresponding derivative MXene phase Mo_2_TiC_2_ has also been widely studied. By preparing Mo_2_TiC_2_ MXene-supported Ru clusters, Wu et al. [17] claimed that it had significant broadband sunlight absorption capacity, high sintering resistance, and good catalytic ability. In addition, Gao et al. [18] also pointed out that Mo_2_TiC_2_ MXene exhibited effective N_2_ affinity and high catalytic activity for electrocatalytic ammonia synthesis.

The above research indicates that the Mo_2_TiAlC_2_ MAX phase and its corresponding etching product Mo_2_TiC_2_ MXene have excellent mechanical and electrical properties. However, the influence of pore structure on such properties in porous materials is also one of the factors that cannot be ignored. Maughan et al. [19] prepared layered porous Mo_2_TiC_2_ MXene by an amine-assisted silicon pillar method, which has a very high specific surface area and enhances the cycling performance of lithium-ion storage. In the research of Yu et al. [20], porous Ni–Cu alloy, which was synthesized by step sintering, exhibited a good electrochemical activation energy of 26.7 kJ mol^−1^. They believed that the microporous structure and the synergistic effect of Ni and Cu jointly contribute to this phenomenon. Wu et al. [21] studied the long-term HER activity of porous Ni_3_Al-Mo in a 6 M KOH solution, and they found that porous Ni_3_Al-Mo electrodes had high surface roughness, which was a key factor for enhancing the hydrogen evolution performance. Zhang et al. [22] prepared porous nickel-titanium alloy using the one-step spark plasma sintering (SPS) method. In their research, as the porosity and pore size increase, the elastic modulus and compressive strength of porous Ni-Ti alloy decrease. This porous material with interconnected pore characteristics, pure phase composition, and low elastic modulus is an ideal candidate for long-term load-bearing hard tissue implants. In our previous study [23], porous Ni-Sn alloy was prepared by activation reaction method and the HER performance was studied. With the increase in Ni content, the porosity showed a trend of first decreasing and then increasing, and the electrochemical active surface area (ECSA) of the electrode also showed the same changing trend. The experimental results revealed that Ni-45%Sn, which had the largest ECSA, displayed the best HER performance. Therefore, this is effective evidence that the pore structure parameter of porous materials is one of the important factors affecting the related electrochemical performance. Given this result, in this article, we do not pay too much attention on the influence of pore structure parameters on electrochemical performance and will focus on the changes in pore structure parameters and the phase transitions.

In the previous study, porous Mo_2_TiAlC_2_ ceramics were prepared by activation reaction sintering method, and the effect of Al content on pore structures was also investigated [24]. Given the excellent performance of Mo_2_TiAlC_2_ in oxidation resistance and other aspects, the introduction of porous structure allows for not only exploring the formation process of Mo_2_TiAlC_2_ from the perspective of phase transition but also observing this process from the perspective of pore structure. What is more, the follow-up product of Mo-based MAX, Mo-based MXene, shows excellent performance in the field of catalysis [17,18,25], and the introduction of porous structure also increases the specific surface area, which can provide more active sites, thus further improve the catalytic activity. In this paper, the effects of different sintering temperatures on pore structure evolutions and phase transformation of the Mo_2_TiAlC_2_ system are discussed. Furthermore, the pore-forming mechanism during the sintering process is described as well.

## 2. Experimental Preparation

**Figure 1:**
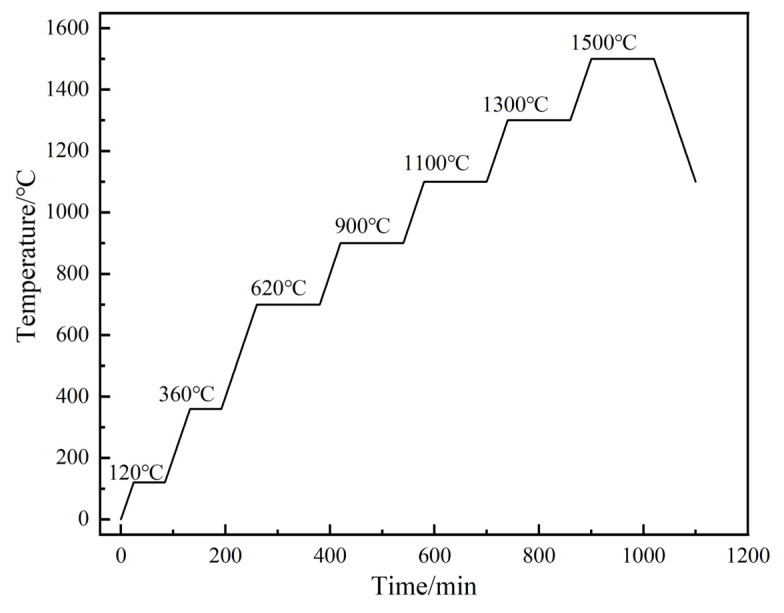
The schematic diagram of sintering procedure.

In the preparation procedure of this experiment, Mo (purity 99.6%), TiH_2_ (purity 99.6%), Al (purity 99.5%) and graphite powder (purity 99.8%) with particle size of 38–74 μm were selected as experimental raw materials, which were mixed at a molar ratio of 2:1:1.3:2. In addition, 5% stearic acid was added into the mixture to facilitate the compression molding. The mixture was subsequently mixed in an omnidirectional planetary ball mill utilizing settings of 400 r/min and an effective mixing length of 6 h, with a 2:1 ratio of zirconia ball to the material. The mixed powders were cold pressed under a pressure of 160 MPa to form Φ25 mm × H2 mm green compacts. Finally, the green compacts were sintered in a vacuum furnace at 1.2 × 10^−3^ Pa under the procedures shown in Figure 1. In order to study the effect of sintering temperature on the evolution of pore structure and phase transformation process of the Mo_2_TiAlC_2_ system, the sintering temperature was gradually increased from 700 °C to 1500 °C, with a temperature increment of 200 °C, and holding each temperature for 2 h. The 120 °C and 360 °C temperatures were held for one hour each to remove the remaining water in the vacuum furnace and stearic acid in the pressed discs.

The size of the round press compacts was measured with vernier calipers before, and after sintering, then the volume expansion was calculated. X-ray diffractometer (XRD; Dmax 2500VB, Rigaku Corp, Tokyo, Japan) was used to analyze the phase transition and composition of porous Mo_2_TiAlC_2_ samples at different sintering temperatures with a scan rate of 2°/min and a step size of 0.02°. Scanning electron microscopy (SEM, Zeiss SIGMA Field Emission SEM, Germany) was introduced to characterize the pore structures of the sintered samples. The bubble point method was utilized to determine pore size [26]. Open porosity and overall porosity were measured by the Archimedes method [27]. On the basis of the standard of DIN EN ISO 4022:2006 [28], the porometer was used to measure nitrogen permeability at 23 °C. In addition, a pore size tester was introduced to measure Mo_2_TiAlC_2_ samples at different sintering temperatures to explore the development law of pore structure.

## 3. Results and Discussion

### 3.1. X-ray Diffraction Analysis (XRD)

**Figure 2:**
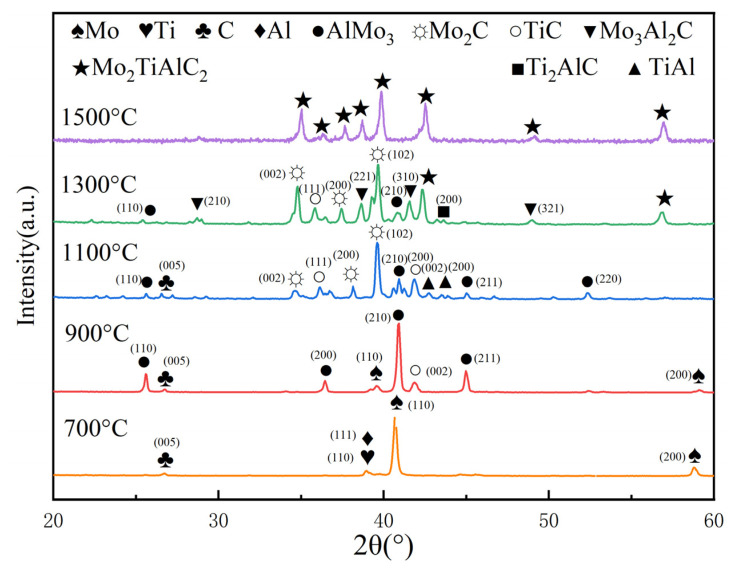
Phase compositions of Mo_2_TiAlC_2_ at different sintering temperatures.

In order to explore the effect of different sintering temperatures on the phase composition of porous Mo_2_TiAlC_2_ ceramics, the sintered specimens ranging from 700 °C to 1500 °C are subjected to XRD analysis, and the tested results are shown in Figure 2. At 700 °C, there are just four single phases (Mo, Ti, Al and C), indicating that TiH_2_ has fully decomposed and that the initial components have not interacted. At a sintering temperature of 900 °C, the AlMo_3_ phase and TiC phase first appears, while the pure Ti and Al phases disappear. At this time, four phases (Mo, AlMo_3_, TiC and C) are present, with the AlMo_3_ phase being the primary phase and the peak intensity of Mo diminishing. Mo_2_C phase and TiAl are clearly visible when the sintering temperature rises to 1100 °C constantly, while the Mo phase is depleted. There are now five phases (Mo_2_C, TiC, AlMo_3_, TiAl and C), with the Mo_2_C and TiC phases serving as the primary phases. When the temperature is further raised to 1300 °C, there are five phases, namely Mo_2_C, TiC, AlMo_3_, Mo_3_Al_2_C and Mo_2_TiAlC_2_. At this point, Mo_2_C, TiC and Mo_3_Al_2_C are the main phases, and the peak strength is enhanced. Concurrently, the C (graphite) phase vanishes, and a large number of Mo_3_Al_2_C and Ti_2_AlC peak phases emerge, along with a small amount of Mo_2_TiAlC_2_ peak phase as minor components. When the temperature rises to 1500 °C, the pure phase Mo_2_TiAlC_2_, whose size of a coherent scattering region is estimated as D_104_ = 63.9 nm, is finally obtained.

In order to conveniently observe the phase transformation of samples from 700 °C to 1500 °C, the phases present at each temperature stage are summarized in Table 1. The reaction processes at various sintering temperatures are analyzed according to the phase changes. At 700 °C, TiH_2_ is completely decomposed, and a titanium phase appears. At this time, the rest of the substances do not react significantly, and the main phases are the elemental powders. Sintering at 900 °C leads to the formation of AlMo_3_, which results from the reaction of melted aluminum with certain amounts of molybdenum, and a small amount of Ti reacts with graphite to generate TiC at the same time. At a sintering temperature of 1100 °C, graphite reacts with molybdenum to form Mo_2_C, while TiAl is formed by the reaction of molten aluminum with a certain amount of titanium. At 1300 °C, Ti_2_AlC is generated by the reaction of TiC and TiAl, while Mo_2_C and MoAl_3_ not only react with each other to yield Mo_3_Al_2_C but also react with graphite to form Mo_2_TiAlC_2_. When the temperature rises to 1500 °C, only the pure substance Mo_2_TiAlC_2_ exists, signifying the complete reaction of all intermediate species to generate Mo_2_TiAlC_2_.

**Figure 3:**
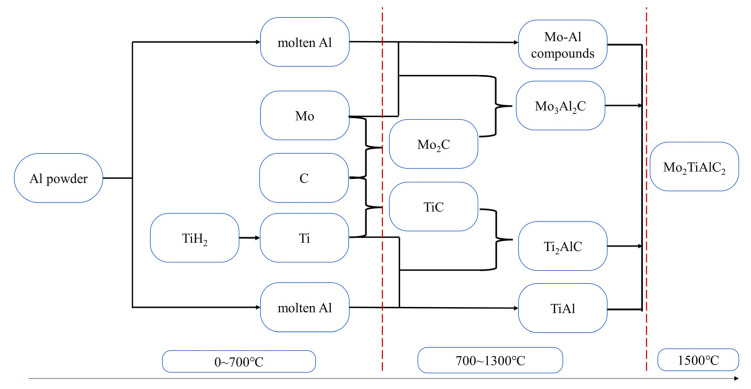
Diagram of the Mo_2_TiAlC_2_ reaction process.

The reaction of Mo_2_TiAlC_2_ with increasing temperature is summarized below, and the reaction process is illustrated in Figure 3.

$$\begin{array}{cc} \left.TiH_{2}=Ti+H_{2}\right\}\;\;\;\;\;\;\;\;\;\;\;\; & 700\;{}^{\circ}C \end{array}$$

$$\begin{array}{c} \left.\begin{array}{c} Al+3Mo=Al{Mo}_{3}\\ Ti+C=TiC \end{array}\right\}\;\;\;\;\;\;\;\;\;\;\;900\;{}^{\circ}C \end{array}$$

$$\begin{array}{c} \left.\begin{array}{c} 2Mo+C={Mo}_{2}C\\ Ti+Al=TiAl \end{array}\right\}\;\;\;\;\;\;\;\;\;\;\;\;1100\;{}^{\circ}C \end{array}$$

$$\begin{array}{c} \left.\begin{array}{c} TiC+TiAl={Ti}_{2}AlC\\ 3{Mo}_{2}C+2TiAl=2{Mo}_{3}{Al}_{2}C\\ {Mo}_{2}C+TiAl+C={Mo}_{2}TiAlC_{2} \end{array}\right\}\;\;\;\;\;\;\;1300\;{}^{\circ}C \end{array}$$

$$\begin{array}{c} \left.\begin{array}{c} 2{Mo}_{3}{Al}_{2}C+3TiAl+4C=3{Mo}_{2}TiAlC_{2}\\ 2AlMo3+3TiC+3C=3{Mo}_{2}TiAlC_{2}\\ {Mo}_{2}C+2AlMo3+2{Ti}_{2}AlC+C=4{Mo}_{2}TiAlC_{2}\\ 3{Mo}_{3}{Al}_{2}C+{Mo}_{2}C+4TiC+C=2{Mo}_{2}TiAlC_{2} \end{array}\right\}\;\;1500\;{}^{\circ}C \end{array}$$

### 3.2. Effects of Temperature on Volume Evolution

**Figure 4:**
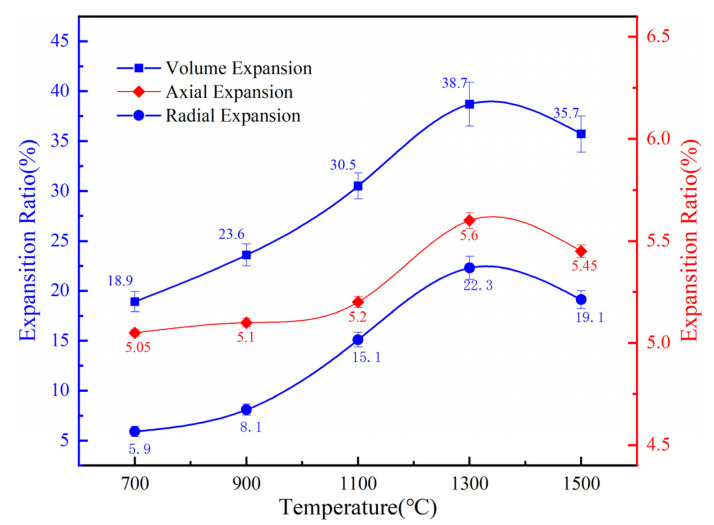
The volume and dimension expansion of the compacts as the temperature rises from 700 to 1500 °C.

The diameter and height of the compact before and after sintering were repeatedly measured by a vernier caliper, and the volume and size changes of the discs were calculated. As shown in Figure 4, with the increase in sintering temperature from 700 °C to 1500 °C, the volume and axial and radial expansion rate of the compact first increase and then decrease. The volume expansion rate keeps growing as the temperature increases from 700 °C to 1300 °C, quickly rising from 18.90% to 38.72%. This phenomenon arises as a result of the fact that when the temperature rises, chemical interactions between substances intensify, intermediate products undergo a constant transformation and an enormous number of pores are subsequently created. When the temperature rises to 1500 °C, the volume expansion rate decreases to 35.74%. This is because the Mo_2_TiAlC_2_ phase is finally formed at this stage and densifies at high temperatures.

### 3.3. Pore Structure Parameters

**Figure 5:**
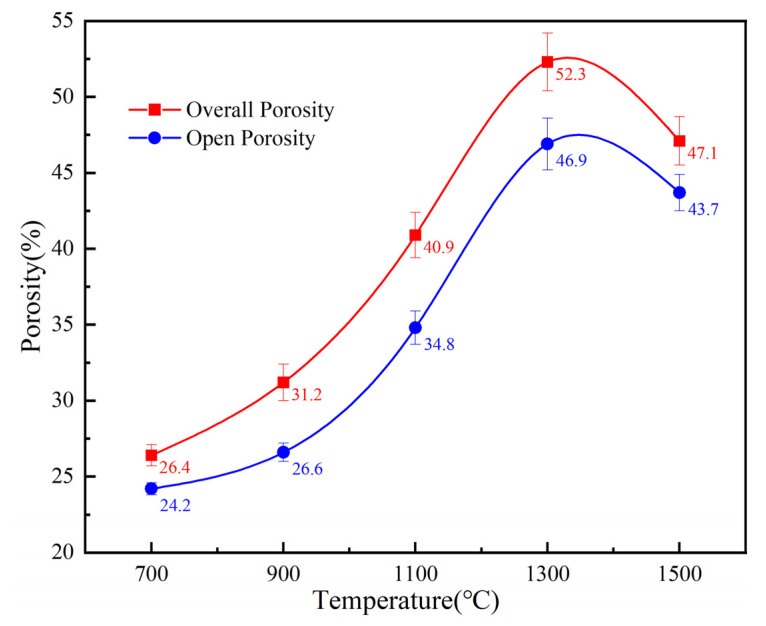
Variation in porosity of the porous Mo_2_TiAlC_2_ with sintering temperature.

Pore structure parameters, including porosity, pore size and permeability, are significant for porous materials; therefore, further exploration helps to study the pore structure and phase changes of porous materials. Figure 5 depicts the trend in pore structural parameters, as it can be seen that the overall porosity is highly similar to open porosity. With the sintering temperature increases from 700 °C to 1300 °C, the porosity increases rapidly from 26.4% to 56.3%. What is worth noting is that the porosity shows a decreasing trend from 1300 °C to 1500 °C, from 56.3% to 47.1%.

**Figure 6:**
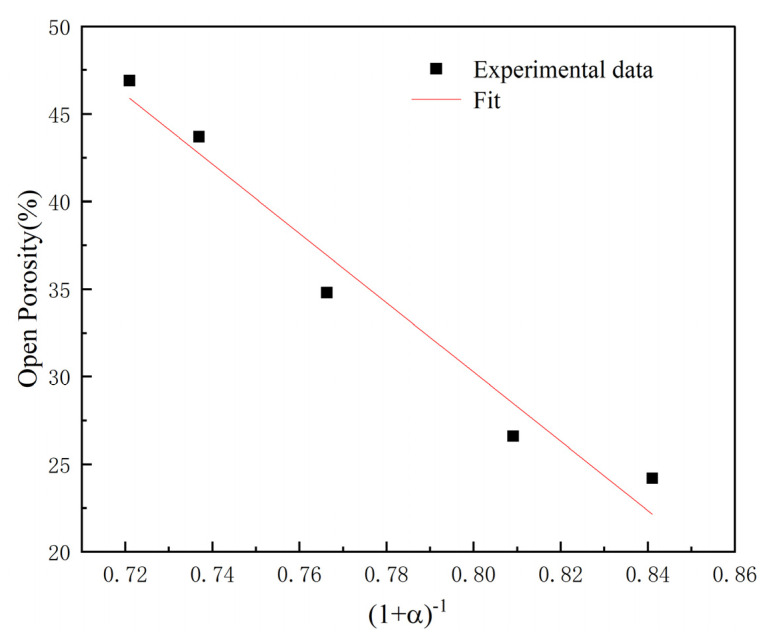
The relationship between the volume expansion ratio and the open porosity obtained by theoretical calculation.

Comparing the trend of volume expansion rate in Figure 4, the change of porosity and expansion rate tends to be consistent. In the research of Jiang. et al. [29], the relationship between the volume expansion and the open porosity can be expressed as:

$$\theta_{p}=\frac{\rho_{0}\left(1-\theta_{0}\right)}{\rho }\frac{1}{1+\alpha }+\left(1-\theta_{c}\right)$$

where $\rho_{0}$ and ρ are the theoretical densities of the compact before and after the sintering, $\theta_{0}$ is the initial overall porosity in green compacts, $\theta_{p}$ is the open porosity after the sintering, α is the volume expansion ratio and $\theta_{c}$ is the closed porosity in the sintered compact. Through the data fitting calculation displayed in Figure 6, it is evident that the trend of change between the two curves is almost proportional.

**Figure 7:**
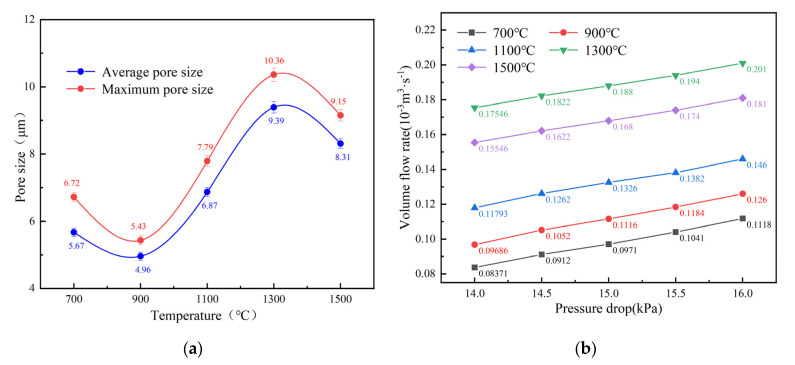
The relationship between the pore structure of Mo_2_TiAlC_2_ porous ceramics and Sintering temperature (**a**) average pore size and maximum pore size, (**b**) nitrogen permeability.

Figure 7a illustrates the relationship between pore size and sintering temperature. At 700 °C, the compact displays a large pore size parameter, which is attributed to the decomposition of TiH_2_. When the temperature reaches 900 °C, the interaction of the slowly diffusing Mo with the melting Al to produce AlMo_3_ causes the pore size to shrink substantially. After that, a considerable number of pores form at their starting locations as a consequence of the reaction of several intermediate products (Mo_2_C, TiC, AlMo_3_, Mo_3_Al_2_C, Ti_2_AlC and Mo_2_TiAlC_2_), and the pore size increases rapidly between 900 and 1300 °C as a result. However, at 1500 °C, the compact exhibits slight densification, leading to shrinkage of internal pores and, finally, a decrease in pore size.

It can be clearly found that the permeability of the sample is linearly correlated with the pressure in Figure 7b. At the same time, the permeability also increases gradually with the increase in temperature and slightly decreases at the final sintering temperature of 1500 °C.

### 3.4. Micromorphology of Mo_2_TiAlC_2_ Ceramic

**Figure 8:**
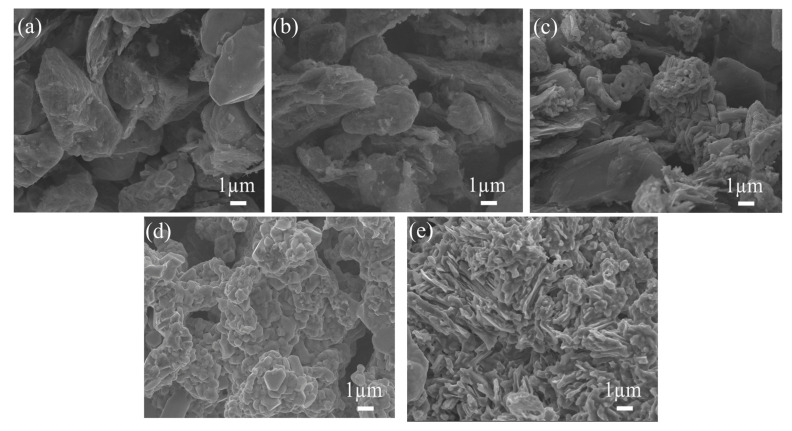
SEM images of samples prepared with different sintering temperatures: (**a**) 700 °C; (**b**) 900 °C; (**c**) 1100 °C; (**d**) 1300 °C; (**e**) 1500 °C.

Figure 8 illustrates the surface morphology of the sintered compacts at different temperatures. It can be seen that at 700 °C, the sample is composed of irregular particles squeezed into each other, and there are a lot of pores between particles. A small amount of layered structure formed on the surface of the sample at 900 °C as a result of the reaction of Mo with melted Al to generate AlMo_3_. When the temperature reaches 1100 °C, the reactions intensify, and particles further connect with one another, resulting in a gradually smooth surface and a more pronounced layer structure. The particles are completely bound at 1300 °C, creating a laminated surface that is neatly polished. The porous Mo_2_TiAlC_2_ ceramic eventually displays a lamellar surface with a substantial number of pores, which show a rather uniform distribution and even pore size at the final sintering temperature of 1500 °C.

### 3.5. Pore Forming Mechanism

From the initial surface morphology, the pressed compact is composed of irregular mixed raw powders, with a large number of retained pores between the powder particles. Combined with the XRD phase constitution analysis, it is obvious that TiH_2_ decomposes completely at 700 °C. At the same time, during the preparation stage, in order to make the mixed powders to be pressed smoothly in the mold, the molding agent stearic acid is added to the mixed powders, which is removed at high temperature, resulting in a significant number of pores as well. As the temperature rises to 900 °C, Mo reacts with melted Al to form AlMo_3_. During the formation of AlMo_3_ intermetallics, due to the diffusion rate of Al being significantly higher than that of Mo [30], the diffusion of Al is dominant, and a large number of pores are produced by the Kirkendall effect [31]. Therefore, when the temperature is lower than 900 °C, the formation of pores is mainly caused by the decomposition of TiH_2_ and the partial diffusion of Al in the Kirkendall effect. Based on Figure 4 and Figure 5, when the temperature rises from 700 °C to 900 °C, the volume expansion rate of the sample increases from 18.9% to 23.6%, with an increase of 29%, while the overall porosity increases from 26.4% to 31.2%, with an increase of 18.2%. At temperatures between 900 °C and 1100 °C, in addition to the TiAl and Mo-Al compounds formed by the Kirkendall effect [32], the formation of carbides TiC and Mo_2_C also promotes the formation of pores. At this stage, the growth rate of the corresponding volume expansion rate is 29.2%, and the overall porosity is 31.1%, both of which are greater than the growth rate from 700 °C to 900 °C. As the temperature continues to rise, between 1100 °C and 1300 °C, with the formation of carbides, MAX also begins to appear at this stage. At this point, the changes in volume expansion rate and overall porosity are 26.9% and 27.9%, respectively, with the variation in porosity still greater than the variation from 700 to 900 °C. Finally, when the temperature rises to 1500 °C, the previously generated intermediate products further participate in the reaction, resulting in the formation of pure phase Mo_2_TiAlC_2_. At the same time, the sample becomes denser, leading to a reduction in pores. Therefore, we can draw a conclusion that in order to control the pore structure of porous Mo_2_TiAlC_2_, controlling the sintering process between 900 and 1300 °C is crucial.

## 4. Conclusions

(1) Porous Mo_2_TiAlC_2_ ceramic is prepared by activation reaction sintering of Mo, Ti, Al and graphite powders at a ratio of 2:1:1.1:2. In addition, the final sintering temperature is 1500 °C, resulting in an expansion rate of 35.74% and a porosity of 47.1%.

(2) The pore-forming mechanism of porous Mo_2_TiAlC_2_ ceramic involves the evolution of the pressed pores, the removal of the molding agent, the decomposition of TiH_2_ and the Kirkendall effect caused by the different diffusion rates of Mo, Ti, Al and C powders and a serious reaction of intermediates during the diffusion process. Firstly, TiH_2_ decomposes at 700 °C. As the temperature progressively rises, the molten Al will combine with Ti and Mo to create TiAl compounds and MoAl compounds, accompanied by the appearance of a small number of carbides. When the temperature continues to rise, the carbides react to the intermetallic complexes TiAl and MoAl to produce the respective intermediate products, Ti-Al-C and Mo-Al-C, which then combine to form the pure Mo_2_TiAlC_2_ at the final sintering temperature of 1500 °C.

## Figures and Tables

**Table 1:** Summary of phase constitution of the samples heated in the different sintering temperatures.

Temperature/°C	Phase Constitution	Major Phase
700	Mo, Ti, Al, C	Single phase
900	Mo, AlMo_3_, TiC, C	AlMo_3_
1100	Mo_2_C, TiC, AlMo_3_, TiAl, C	Mo_2_C, TiC
1300	Mo_2_C, TiC, AlMo_3_, Mo_3_Al_2_C, Ti_2_AlC, Mo_2_TiAlC_2_	Mo_2_C, TiC, Mo_3_Al_2_C
1500	Mo_2_TiAlC_2_	Mo_2_TiAlC_2_

## Data Availability

Not applicable.

## References

[1] Azina C., Tunca B., Petruhins A., Xin B., Yildizhan M., Persson P.O.Å., Vleugels J., Lambrinou K., Rosen J., Eklund P. (2021). Deposition of MAX phase-containing thin films from a (Ti,Zr)_2_AlC compound target. Appl. Surf. Sci..

[2] Azzouz-Rached A., Hadi M.A., Rached H., Hadji T., Rached D., Bouhemadou A. (2021). Pressure effects on the structural, elastic, magnetic and thermodynamic properties of Mn_2_AlC and Mn_2_SiC MAX phases. J. Alloys Compd..

[3] Badie S., Sebold D., Vaßen R., Guillon O., Gonzalez-Julian J. (2021). Mechanism for breakaway oxidation of the Ti_2_AlC MAX phase. Acta Mater..

[4] Ghasali E., Derakhshandeh M.R., Orooji Y., Alizadeh M., Ebadzadeh T. (2021). Effects of 211 and 413 ordering on the corrosion behavior of V-Al-C MAX phases prepared by spark plasma sintering. J. Eur. Ceram. Soc..

[5] Hadi M.A. (2020). Superconducting phases in a remarkable class of metallic ceramics. J. Phys. Chem. Solids.

[6] Rud A.D., Lakhnik A.M., Kirian I.M., Sizonenko O.N., Zaychenko A.D., Pristash N.S., Rud N.D. (2018). Mechanochemical synthesis and structure of metal-carbon composites based on the MAX phases. Mater. Today: Proc..

[7] Hossein-Zadeh M., Ghasali E., Mirzaee O., Mohammadian-Semnani H., Alizadeh M., Orooji Y., Ebadzadeh T. (2019). An investigation into the microstructure and mechanical properties of V_2_AlC MAX phase prepared by microwave sintering. J. Alloys Compd..

[8] Bhattacharya R., Goulbourne N.C. (2016). Heterogeneous strain evolution in representative polycrystalline MAX phases. Int. J. Solids Struct..

[9] Tallman D.J., Naguib M., Anasori B., Barsoum M.W. (2012). Tensile creep of Ti_2_AlC in air in the temperature range 1000–1150 °C. Scr. Mater..

[10] Liu Z., Wu E., Wang J., Qian Y., Xiang H., Li X., Jin Q., Sun G., Chen X., Wang J. (2014). Crystal structure and formation mechanism of (Cr_2/3_Ti_1/3_)_3_AlC_2_ MAX phase. Acta Mater..

[11] Caspi E.a.N., Chartier P., Porcher F., Damay F., Cabioc’h T. (2014). Ordering of (Cr,V) Layers in Nanolamellar (Cr_0.5_V_0.5_)_n+1_AlC_n_ Compounds. Mater. Res. Lett..

[12] Zou H., Li X., Zhang C., Wen Y., Fan Y., Liu Y., Xiong L., Zheng X., Yang J. (2021). Reactive synthesis for porous TiVAlC ceramics by TiH_2_,V, Al and graphite powders. Ceram. Int..

[13] Anasori B., Halim J., Lu J., Voigt C.A., Hultman L., Barsoum M.W. (2015). Mo_2_TiAlC_2_: A new ordered layered ternary carbide. Scr. Mater..

[14] Qing-He G., Zhi-Jun X., Ling T., Xianjun Z., Guozhu J., An D., Rong-Feng L., Yun-Dong G., Ze-Jin Y. (2016). Evidence of the stability of Mo_2_TiAlC_2_ from first principles calculations and its thermodynamical and optical properties. Comput. Mater. Sci..

[15] Anasori B., Dahlqvist M., Halim J., Moon E.J., Lu J., Hosler B.C., Caspi E.a.N., May S.J., Hultman L., Eklund P. (2015). Experimental and theoretical characterization of ordered MAX phases Mo_2_TiAlC_2_ and Mo_2_Ti_2_AlC_3_. J. Appl. Phys..

[16] Hadi M.A., Ali M.S. (2016). New ordered MAX phase Mo_2_TiAlC_2_: Elastic and electronic properties from first-principles. Chin. Phys. B.

[17] Wu Z., Shen J., Li C., Zhang C., Feng K., Wang Z., Wang X., Meira D.M., Cai M., Zhang D. (2022). Mo_2_TiC_2_ MXene-Supported Ru Clusters for Efficient Photothermal Reverse Water-Gas Shift. ACS Nano.

[18] Gao Y., Cao Y., Zhuo H., Sun X., Gu Y., Zhuang G., Deng S., Zhong X., Wei Z., Li X. (2020). Mo_2_TiC_2_ MXene: A Promising Catalyst for Electrocatalytic Ammonia Synthesis. Catal. Today.

[19] Maughan P.A., Bouscarrat L., Seymour V.R., Shao S., Haigh S.J., Dawson R., Tapia-Ruiz N., Bimbo N. (2021). Pillared Mo_2_TiC_2_ MXene for high-power and long-life lithium and sodium-ion batteries. Nanoscale Adv..

[20] Yu L., Lei T., Nan B., Jiang Y., He Y., Liu C.T. (2016). Characteristics of a sintered porous Ni–Cu alloy cathode for hydrogen production in a potassium hydroxide solution. Energy.

[21] Wu L., He Y., Lei T., Nan B., Xu N., Zou J., Huang B., Liu C.T. (2014). The stability of hydrogen evolution activity and corrosion behavior of porous Ni_3_Al–Mo electrode in alkaline solution during long-term electrolysis. Energy.

[22] Zhang L., Zhang Y.Q., Jiang Y.H., Zhou R. (2015). Superelastic behaviors of biomedical porous NiTi alloy with high porosity and large pore size prepared by spark plasma sintering. J. Alloys Compd..

[23] Yang J., Li J., Wang Y., Dong S., Fan Y., Liu W., Kuang Y., Tan S., Xiao G., Wang B. (2022). Tailoring the Pore Structure of Porous Ni-Sn Alloys for Boosting Hydrogen Evolution Reaction in Alkali Solution. Metals.

[24] Yang J., Wen Y., Li X., Fan Y., Zou H., Zhang C., Xiong L., Liu Y. (2022). The influence of Al content on pore structures of porous Mo_2_TiAlC_2_ ceramics through powder metallurgy technique. J. Asian Ceram. Soc..

[25] Li G., Zhou B., Wang P., He M., Fang Z., Yuan X., Wang W., Sun X., Li Z. (2022). High-Efficiency Oxygen Reduction to Hydrogen Peroxide Catalyzed by Oxidized Mo_2_TiC_2_ MXene. Catalysts.

[26] DIN EN ISO 4022:2006. Permeable Sintered Metal Materials—Determination of Bubble Test Pore Size. https://webstore.ansi.org/standards/din/dineniso40222006.

[27] Liu X., Zhang H., Jiang Y., He Y. (2015). Characterization and application of porous Ti_3_SiC_2_ ceramic prepared through reactive synthesis. Mater. Des..

[28] Zhou Y., Sun Z. (2000). Electronic structure and bonding properties of layered machinableTi_2_AlC and Ti_2_AlNceramics. Phys. Rev. B.

[29] Jiang Y., He Y.H., Xu N.P., Zou J., Huang B.Y., Liu C.T. (2008). Effects of the Al content on pore structures of porous Ti–Al alloys. Intermetallics.

[30] Neumann G., Tuijn C. (2008). Self-Diffusion and Impurity Diffusion in Pure Metals.

[31] He Y.H., Jiang Y., Xu N.P., Zou J., Huang B.Y., Liu C.T., Liaw P.K. (2007). Fabrication of Ti–Al Micro/ Nanometer-Sized Porous Alloys through the Kirkendall Effect. Adv. Mater..

[32] Yang J., Liao C., Wang J., Jiang Y., He Y. (2014). Effects of the Al content on pore structures of porous Ti_3_AlC_2_ ceramics by reactive synthesis. Ceram. Int..

